# Behçet's Syndrome as a Model of Thrombo-Inflammation: The Role of Neutrophils

**DOI:** 10.3389/fimmu.2019.01085

**Published:** 2019-05-14

**Authors:** Giacomo Emmi, Matteo Becatti, Alessandra Bettiol, Gülen Hatemi, Domenico Prisco, Claudia Fiorillo

**Affiliations:** ^1^Department of Experimental and Clinical Medicine, University of Firenze, Firenze, Italy; ^2^Department of Experimental and Clinical Biomedical Sciences “Mario Serio”, University of Firenze, Firenze, Italy; ^3^Department of Neurosciences, Psychology, Pharmacology and Child Health (NEUROFARBA), University of Firenze, Firenze, Italy; ^4^Division of Rheumatology, Department of Internal Medicine, Istanbul University – Cerrahpaşa, Istanbul, Turkey

**Keywords:** Behçet's syndrome, thrombosis, neutrophils, oxidative stress, fibrinogen

## Abstract

Behçet's syndrome (BS) is a systemic vasculitis, clinically characterized by different organ involvement and often complicated by thrombosis which occurs in vessels of all sizes. Thrombosis is more frequent in male patients with active disease and represents an important cause of morbidity and mortality. Neutrophil involvement in BS has been repeatedly suggested in the last few years. Indeed, neutrophils have been shown to be hyperactivated in BS patients, probably with a HLAB51 related contribution, and represent the main cells infiltrating not only oral and genital ulcers or erythema nodosum, but also other sites. Besides being deputed to host defense against micro-organisms, neutrophils display fundamental roles both in inflammation and tissue damage becoming inappropriately activated by cytokines, chemokines and autoantibodies and subsequently producing large amounts of superoxide anion (${\text{O}}_{2}^{.}$) via NADPH oxidase (NOX2). The strict relationship between inflammation and hemostasis has been already demonstrated. Indeed, inflammation and immune-mediated disorders increase the risk of thrombosis, but the pathways that link these processes have not been completely elucidated. In this regard, we recently demonstrated, in a large population of BS patients, a new neutrophil-dependent pathogenetic mechanism of thrombosis. In particular, it was shown that neutrophils, mainly through NADPH oxidase, produce excessive amounts of reactive oxygen species (ROS), which are able to markedly modify the secondary structure of fibrinogen and hence the overall architecture of the fibrin clot that becomes less susceptible to plasmin-induced lysis. These data point out that BS represents “*per se*” a model of inflammation-induced thrombosis and suggest that neutrophils specifically contribute to thrombo-inflammation in this rare disease. In particular, it is suggested that an alteration in fibrinogen structure and function are associated with enhanced ROS production via neutrophil NADPH oxidase. Altogether, these findings improve our understanding of the intricate pathogenetic mechanisms of thrombo-inflammation and may indicate potential new therapeutic targets.

## Introduction

Behçet's syndrome (BS) is a peculiar disease of unknown origin (1). During the years, BS has been classified among spondyloarthropathies (2), autoinflammatory disorders (3), and more recently as a systemic vasculitis (4). However, BS is considered by some authors a neutrophilic dermatosis, not only for the muco-cutaneous aspects, but mainly for the presence of an intense neutrophilic infiltrate at histological level (5). Indeed, neutrophils are the main cells infiltrating not only oral and genital ulcers or erythema nodosum, but also other sites (i.e., eyes, central nervous system, vessel wall) (6, 7).

In recent years, neutrophils have also emerged as contributors of the initiation/progression of thrombotic events, both in veins and arteries (8). Indeed, three main mechanisms have been described so far by which neutrophils can contribute to thrombo-inflammation in either inflammatory or neoplastic conditions: (1) by the release of neutrophil extracellular traps (NETs); (2) by the formation of neutrophil-derived microparticles; and (3) via the hyperactivation of inflammasome. Interestingly, in BS the contribution of microparticles and inflammasome has been already suggested (9, 10), while the role of NETs is still under investigation.

Recently, we demonstrated in BS patients, a new mechanism by which neutrophils can induce and favor thrombosis. Neutrophils, mainly through NADPH oxidase, are able to produce huge amounts of reactive oxygen species (ROS), so modifying the secondary structure of fibrinogen, that becomes less susceptible to plasmin-induced fibrinolysis (11).

In this review we will briefly describe the structure of NAPDH oxidase and we will show the main evidence about the ability of neutrophils to induce damage through this enzymatic platform. After that, we will describe the toxic role of ROS and the potential correlation with NET formation. Finally, we will briefly depict the already known role of neutrophils in BS, connecting it to the new mechanism that we recently described in BS patients, by which neutrophils significantly contribute to thrombo-inflammation in this pathologic condition.

## Neutrophils and NADPH Oxidase

Neutrophils, which are also defined as polymorphonuclear leukocytes, are phagocytic cells of the innate immune system deputed to host defense against micro-organisms, the microbicidal mechanism, centered on ROS and proteolytic enzymes, occurs within the phagolysosomes, the amalgam of phagosomes and lysosomes (12). Neutrophils also display fundamental roles in inflammation and tissue injury in inflammatory diseases, becoming improperly activated by different molecules, namely cytokines, chemokines, and autoantibodies (13). Upon activation, neutrophils produce large amounts of superoxide anion (${\text{O}}_{2}^{-.}$) via NADPH oxidase (NOX2), an enzyme complex which, using NADPH as the electron donor and flavin-adenine dinucleotide (FAD), catalyzes the monovalent reduction of oxygen (14). So far, seven homologs of NADPH oxidase (NOX1–NOX5 and Duox1 and 2) have been described (15). All NADPH oxidases, with the exception of NOX5, Duox1 and 2, are characterized by a similar topological structure of the catalytic core of gp91phox. NOX1, NOX2, and NOX4 are mainly expressed in the vascular system, and are strongly involved in inflammation-induced vascular injury (16).

**Figure 1:**
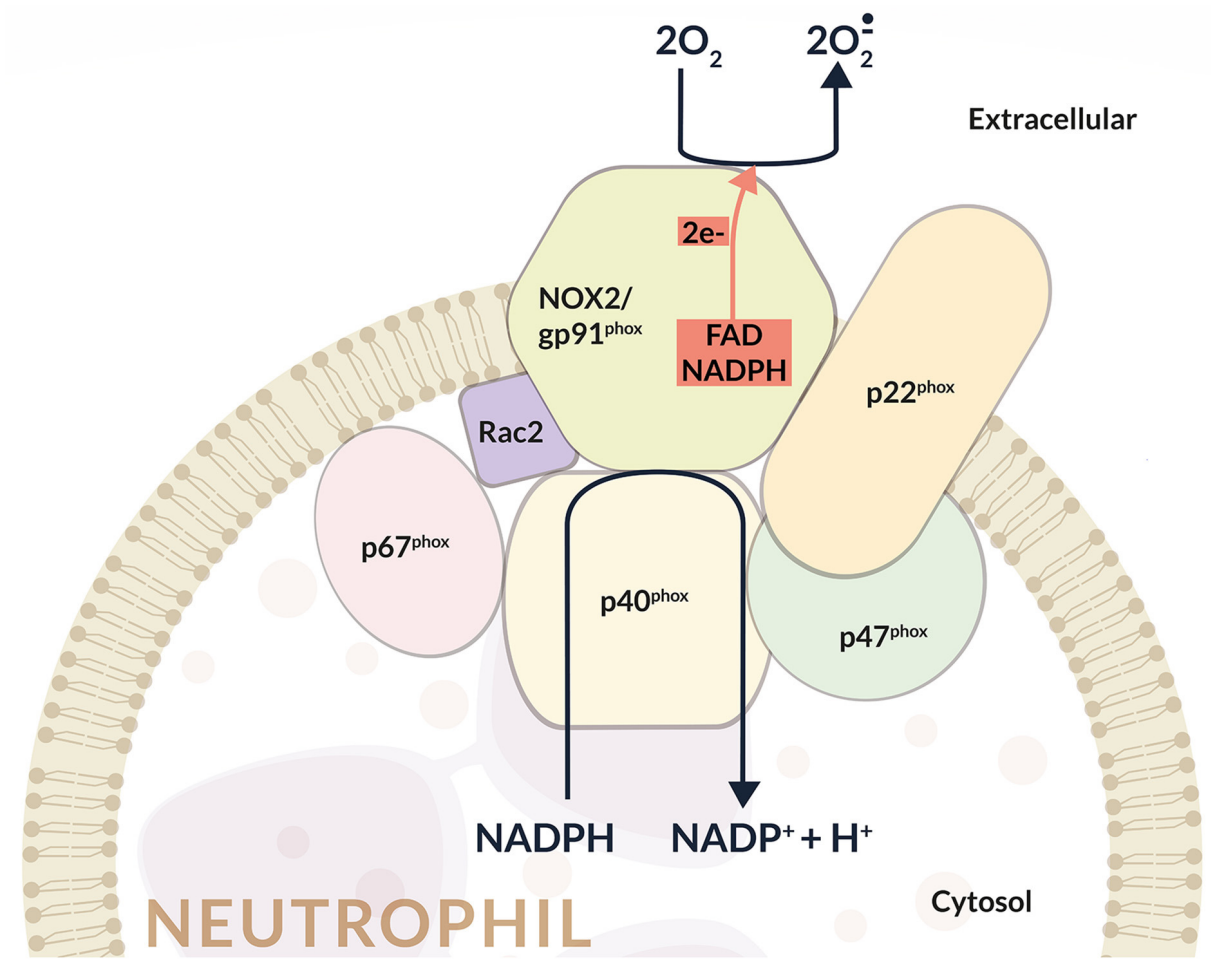
In BS, reactive oxygen species (ROS) are released by different cell types, and mainly by neutrophils through a NADPH-oxidase dependent mechanism. Proteins represent main ROS targets. In this context ROS can exert deleterious effects on fibrinogen, affecting its biological activity upon oxidation. Fibrinogen oxidation leads to significantly marked fibrinogen secondary structure alterations and hence a more thrombogenic clot with a tight network characterized by thin fibrin fibers that result more resistant to plasmin-induced lysis. This complex neutrophil-mediated mechanism, significantly contributes to thrombo-inflammation.

NOX2 activation depends on the assembly of four cytosolic proteins (p47phox, p67phox, p40phox, and Rac2) with two transmembrane proteins p22phox and gp91phox, which constitute the flavocytochrome b558 complex, the catalytic core of NOX2. In particular, the gp91phox subunit is formed by six transmembrane domains, and in its C-terminal region are situated the binding sites for FAD and NADPH (Figure 1).

The association of the cytosolic subunits with the membrane-bound flavocytochrome b558 complex is responsible for the transfer of electrons from cellular NADPH to molecular oxygen and the subsequent ${\text{O}}_{2}^{-.}$ production. Once activated, about 10 nmol/min ${\text{O}}_{2}^{-.}$ per million neutrophils are produced through the so called oxidative burst (17) (Figure 1).

This multicomponent enzyme is finely regulated to guarantee activation when required, and is inhibited when ROS production must be restricted. At present, all the subunits that compose NADPH oxidase have been completely identified and also the mechanisms that regulate their activation have been characterized. In this regard, it has been shown that during the assembly of the oxidase complex, p47phox phosphorylation and Rac activation are essential. In particular, upon phosphorylation, p47phox auto-inhibitory conformation is removed allowing the binding to p22phox. Pro-inflammatory cytokines (TNF-α, GM-CSF, and G-CSF), LPS, phorbol-12-myristate-13-acetate (PMA), and N-formylmethionyl leucyl phenylalanine (fMLP) display fundamental roles in p47phox phosphorylation (18, 19).

p47phox has been considered fundamental in the progression of several pathological processes. Indeed, in p47phox null mice the formation of atherosclerotic lesions and development of pulmonary fibrosis as well as the TNF-α-induced lung inflammation or sepsis-induced lung microvascular injury were dramatically reduced (20, 21).

The activity of NOX2 has also been evoked in models of cardiac inflammation and fibrosis. Indeed, in several experimental models of NOX2 deficient mice [induced by coronary artery ligation (22), aortic banding (23), Ang II infusion (24), or doxorubicin treatment (25)] cardiac remodeling and contractile dysfunction were markedly reduced compared with the wild-type mice.

Moreover, in aortic banding model, N-acetylcysteine treatment of wild-type mice resulted in recovery of contractile dysfunction (23). All these findings suggest the fundamental role of NOX2-derived ROS in cardiac remodeling and the preventive role of antioxidants.

Rac1 or Rac2 GTPase have been suggested to be essential for oxidase activity in cell-free systems (26, 27). Rac2, the predominantly active isoform in human neutrophils seems to represent an integral and required component of the NADPH oxidase in the intact leukocyte (28). Importantly, it has also been demonstrated that rac2^−^ neutrophils display reduced or absent superoxide production in response to various stimuli (29–31). Rac2 translocates to the plasma membrane phospholipid bilayer via its prenylated C-terminus and polybasic domain. It has been suggested that Rac insert domain may directly interact with cytochrome b558. Having said that, different models exist to explain the oxidase regulation by Rac GTPase: adapter vs. active participant and it is not clear how all the experimental data that support each model can be reconciled (32, 33).

Interestingly, several studies are currently underway to identify the pathways involved in NADPH oxidase regulation in order to develop novel strategies to regulate ROS production by neutrophils in inflammatory diseases (34).

NADPH oxidase-derived ROS have a role both in microbial killing and in innate immunity; however, excessive ROS production by neutrophils at the site of inflammation is responsible for harmful effects such as endothelial dysfunction and tissue injury. Specifically, during inflammation, neutrophil-dependent oxidative stress induces the opening of inter-endothelial junctions and the movement of inflammatory cells across the endothelial barrier. The migrated inflammatory cells are effective in the elimination of pathogens and foreign particles, but are also responsible for tissue injury. Thus, particular attention should be paid to NADPH oxidase hyperactivation and to consequent enhanced ROS production.

Neutrophils can easily produce ROS and proteases causing both damage to host tissues and, modifications of host proteins, lipids and DNA with alteration in redox homeostasis (13). Commonly, this injury can be resolved by resident macrophages, which are able to remove apoptotic neutrophils and injured tissue as part of the normal process of inflammation resolution (35).

## The Dual Role of ROS

In healthy condition, a balance between oxidant formation and endogenous antioxidant defense mechanisms exists. When this equilibrium is disturbed, oxidative stress and associated damage is developed. Particularly, all cellular components such as DNA, proteins, and membrane lipids are injured and cell death may occur. These events are responsible for the onset of several pathological conditions including diabetes, cardiovascular diseases, cancer, degenerative diseases, and chronic inflammatory disorders (15).

To prevent the harmful effects of oxidants, cells have developed an antioxidant defense systems specifically targeted to ROS removal. The antioxidant enzymes superoxide dismutase (SOD), catalase, glutathione peroxidase, peroxiredoxins, glutaredoxins, and thioredoxins represent the first line of defense against ROS attack. Other non-enzymatic antioxidant compounds are able to directly react with oxidizing agents and disarm them. Such antioxidants (vitamin E, vitamin C, uric acid, glutathione, and ceruloplasmin) are “scavengers,” and their role is unavoidably suicidal (36).

Among oxidant molecules, ${\text{O}}_{2}^{-.}$ is a very unstable molecule which can rapidly undergo dismutation either spontaneously or via enzymatic reaction with superoxide dismutase (SOD) into hydrogen peroxide (H_2_O_2_). Another important event that commonly occurs at vascular level is the reaction between ${\text{O}}_{2}^{-.}$ and nitric oxide (which possesses strong anti-inflammatory properties) with the consequent formation of the highly reactive molecule peroxynitrite (ONOO^−^) (14). Hydrogen peroxide easily crosses the membranes, is microbicidal at high concentrations, and represents the major substrate of myeloperoxidase (MPO). This is a neutrophil heme peroxidase stored within the azurophilic neutrophil granules and responsible for the generation of the powerful bactericidal hypochlorous acid (HClO), which, together with proteases, generates a highly cytotoxic intra-phagosomal environment able to eliminate micro-organisms, but also to induce strong chemical modification of proteins, lipids and DNA (36). HClO generation by MPO, is critical in the production of neutrophil extracellular traps (NETs) and is implicated in DNA damage and histone modification related with inflammatory disease (37).

At low concentrations, ROS display signaling effects in both intra and extra-cellular signal transduction pathways, by regulating several cellular processes, such as proliferation, survival, differentiation, and metabolism (38). At intracellular level, specific enzymes, the mitogen-activated protein kinases (MAPK), are responsive to ROS in a cell type- and stimulus-specific manner (39). Endogenous H_2_O_2_ production by neutrophils is tightly regulated by p38-MAPK and c-Jun n-terminal kinase (JNK) and is responsible for ERK1/2 phosphorylation (40).

During inflammatory response, leukocytes and mast cells, which are present in the injured areas, produce a “respiratory burst” because of an enhanced uptake of oxygen and therefore overproduce and release ROS at the damaged area. However, inflammatory cells produce more soluble inflammatory mediators such as cytokines, arachidonic acid, and chemokines, which act through active inflammatory cells in the affected area and release more reactive species (41).

Neutrophil ROS are specifically involved in migration to and from wounds and play significant role in the resolution of neutrophil-mediated inflammation. ROS environment also regulates adhesion molecule expression. Neutrophil adhesion to the surface of endothelial cells, and subsequent extravasation into tissues, implicates ROS-induced phosphorylation of specific molecular targets (42). In this regard, it has been demonstrated that treatment of human umbilical vein endothelial cells (HUVEC) with H_2_O_2_ or t-butylhydroperoxide resulted in transcriptional independent surface translocation of P-selectin, leading to increased neutrophil adherence.

## Neutrophils and NET Formation

NETs are neutrophil-derived structures mainly composed of DNA and histones, also comprising MPO and other microbicidal proteins, produced in response to infectious or inflammatory stimuli. NETs represent an alternative defense mechanism used by neutrophils to trap and kill micro-organisms (43).

Recently, venous thrombosis has been associated to neutrophil activation and NETs release. The role of NETs in thrombosis has been established by translational animal venous thrombosis models (44) and human studies (45). Both *in vivo* thrombosis models and genetically modified mice have allowed the uncovering of the specific role of NETosis in the pathogenesis of thrombosis. Importantly, neutrophil activation and NETosis, linked to acute infection/inflammation, is also able to induce thrombosis. This mechanism of thrombo-inflammation has been proposed to be involved not only in the pathogenetic mechanisms of sepsis, but also of autoimmune diseases (46) and of cancer (47).

In particular, it has been found that some NET proteins, in particular histones, are post-translationally modified, by methylation, acetylation, and citrullination, thus suggesting that NETs may represent a source of auto-antigens in autoimmune disease (48). A strict link between NETosis and NOX2 has been witnessed in neutrophils from patients affected by chronic granulomatous disease (a disease characterized by a genetic defect preventing assembly of a functional NADPH oxidase) which produce neither ROS nor NETs (49).

ROS regulate several processes of NET formation, from the induction of morphological changes to the increase in membrane permeability and the release of neutrophil elastase from granules, thus inducing the degradation of histones and chromatin decondensation. ROS are also able to inhibit apoptosis and promote autophagy, a process of lysosome-mediated intracellular degradation permitting protein turnover (50). In particular, it has been shown that ROS level determines whether autophagy reactions lead to NETosis (51).

However, the implication of NETS and NETosis in the pathophysiology of BS, despite being recently suggested by some authors, has not been definitely demonstrated (52, 53).

## Neutrophils and Behçet: an Old *liaison*

Neutrophils are hyperactivated in BS patients, probably with a HLAB51 related contribution (54). Matsumura and Mizushima have firstly suggested this concept about 40 years ago, in a pioneering study (55). However, the potential role of neutrophils in the immunopathogenesis of the disease has been suggested by different direct and indirect evidence (1). Typically, BS shows a different histology compared to other systemic vasculitis. Indeed, some manifestations of BS such as mucocutaneous and central nervous system lesions can be considered a neutrophilic perivasculitis, rather than a true vasculitic process (7, 56). Neutrophils accumulate around the *vasa vasorum* not only of the large vessel wall (57), but a dense neutrophilic infiltration can also be found at cutaneous (57), articular (58), ocular (59), intestinal (60), and neurological level (61). It has been also suggested that different adhesion molecules enhancing the adhesion of neutrophils to endothelial cell (62, 63), contribute to the neutrophilic infiltration in BS.

In BS the inflammatory activity of neutrophils is probably orchestrated by the release of different chemokines and cytokines, and in particular CXCL8 (previously known as IL-8) and IL-17. Indeed, in BS patients skin-derived T-cell clones are able to release large amounts of CXCL-8, a potent chemoattractant of neutrophils. Moreover, serum levels of CXCL-8 are high in the active phase of the disease (64). Different studies have suggested that several cell types are able to produce IL-17 in BS patients (65–67), thus suggesting again a pivotal role of neutrophils in the propagation of the inflammation in this condition. Recently, the capacity of testosterone to activate neutrophils has been suggested, thus partly accounting for the more aggressive course of the disease among men (68).

During thrombosis, several inflammatory and thrombogenic signals display synergistic effects resulting in platelets-leukocytes interaction finally developing in aggregate formation (69, 70). Platelets induce neutrophil activation which produce oxidants and cytokines: neutrophil-platelet aggregates are involved in the maintenance and the amplification of the systemic inflammatory response. Neutrophils/platelets aggregates exert inflammatory and also thrombogenic effects representing common events of both acute cardiovascular diseases and of systemic inflammatory, neoplastic, and autoimmune diseases (71).

## Neutrophils, ROS, and Inflammation Induced-Thrombosis in Behçet

Inflammation and hemostasis or thrombosis have been shown to represent integrated processes.

Indeed, inflammation and autoimmune disorders are also associated to an increased risk of thrombosis (72), but the pathways that link these processes are far to be elucidated.

BS is a systemic vasculitis with multi-system involvement, including mouth, eyes, genitals and brain. The disease is often complicated by thrombosis which predominantly affects male patients with active disease and represents an important cause of morbidity and mortality (73).

Vessels of all sizes are affected in the disease and the most common vascular manifestations are represented by deep and superficial vein thrombosis of the lower extremities. In the onset of thrombotic events, systemic inflammation, which seems to play a fundamental role in BS more than usual thrombophilic factors, has been shown to be mediated by T lymphocytes, monocytes, neutrophils, and proinflammatory cytokines along with endothelial cell dysfunction (74).

The understanding of the above pathogenetic mechanisms, together with the clinical experience, has led to consider thrombosis in BS as inflammatory–mediated and consequently, has suggested its treatment with immunosuppression rather than anticoagulation (7, 56, 75). However, the specific details of the complex crosstalk between inflammation and hemostasis are not completely understood (76).

Notably, the array of processes related to inflammatory thrombosis comprises pathways that are not fully responsive to anti-thrombotic management (directed at the extrinsic pathway and generation of thrombin) (76). It is now widely accepted that a strict relationship among inflammation, endothelial dysfunction and oxidative stress exists (15). In particular, neutrophils enhance the increased risk, severity and adverse outcome of thrombosis acting as modulator of several processes: causing the rupture of atherosclerotic plaque, inducing platelet activation, possible tissue factor carriage, altering the antithrombotic function of the endothelium and inhibiting response to fibrinolytic agents (6, 77, 78). In addition, a dose-dependent relationship between neutrophil activation, circulating nucleosomes and development of deep vein thrombosis has been reported (79).

Indeed, a global blood redox dishomeostasis (revealed by ischemia-modified albumin, advanced oxidation protein products, and overall pro-oxidant/antioxidant balance) has been reported in BS patients (80). Specifically, lipid peroxidation markers in serum, erythrocytes and neutrophils and decreased levels of antioxidant enzymes (glutathione peroxidase, catalase) have been reported in BS patients and have been even indicated as prognostic tools in this disease (81).

Serum from BS patients also exhibits increased ROS levels, mainly represented by ${\text{O}}_{2}^{-.}$ and H_2_O_2_ and clear signs of NETosis (82). Enhanced levels of plasma MPO activity have been found in BS patients, besides to raised levels of plasma nitrate/nitrite, which are substrates for MPO and induce the reactive nitrogen dioxide (NO_2_) oxidizing agent generation (83). MPO-dependent nitrate/nitrite depletion, leads to the reduction of these substrates for nitric oxide synthase reactions and consequent decrease in the production of nitric oxide (NO) a crucial modulator of smooth muscle contraction and vasodilation (84).

In inflammation, besides autoimmunity and coagulation, fibrinogen, a plasma protein particularly susceptible to oxidation, plays key roles (85). The ability of fibrinogen to contribute to the inflammatory response rely on its specific interaction with integrins, which are leukocyte cell surface adhesion receptors expressed on neutrophils, monocytes, macrophages, and several subsets of lymphocytes (86). Accordingly, some authors recently showed neutrophil hyperfunction, increased ROS production and endothelial cell dysfunction associated to an impaired fibrinolysis in BS patients (87).

To clarify the possible relationship among these processes, a recent study by our group, performed in a large population of BS patients, was undertaken to elucidate the mechanisms of inflammation-induced thrombosis. In that study, fibrinogen oxidative modifications, fibrinogen protein structure, fibrinogen function (assessed in terms of thrombin-dependent fibrin polymerization and fibrin susceptibility to plasmin-induced lysis) and possible blood ROS sources were explored This latter aspect must be fully considered because only few studies have put the attention on the potential blood ROS sources in BS (83).

In the reported experiments, BS patients showed a global redox status impairment along with an enhanced fibrinogen carbonylation. Furthermore, fibrinogen polymerization and fibrin susceptibility to plasmin-induced lysis was markedly affected in patients when compared to control subjects.

In another series of experiments aimed to clarify the mechanism of fibrinogen oxidation, it was shown that purified fibrinogen was markedly carbonylated when incubated with neutrophils derived from BS patients, but not with monocytes or lymphocytes from the same patients. This went along with a significant increase in NADPH oxidase activity which was specifically evident only in the neutrophil fraction. Importantly, in Behçet patients, the extent of fibrinogen oxidation appeared significantly correlated with neutrophil-derived ROS, but not with lymphocyte- or monocyte-derived ROS.

Fibrinogen oxidation was also strictly related to fibrinogen function which resulted significantly affected both in terms of polymerization and in terms of plasmin-induced lysis.

**Figure 2:**
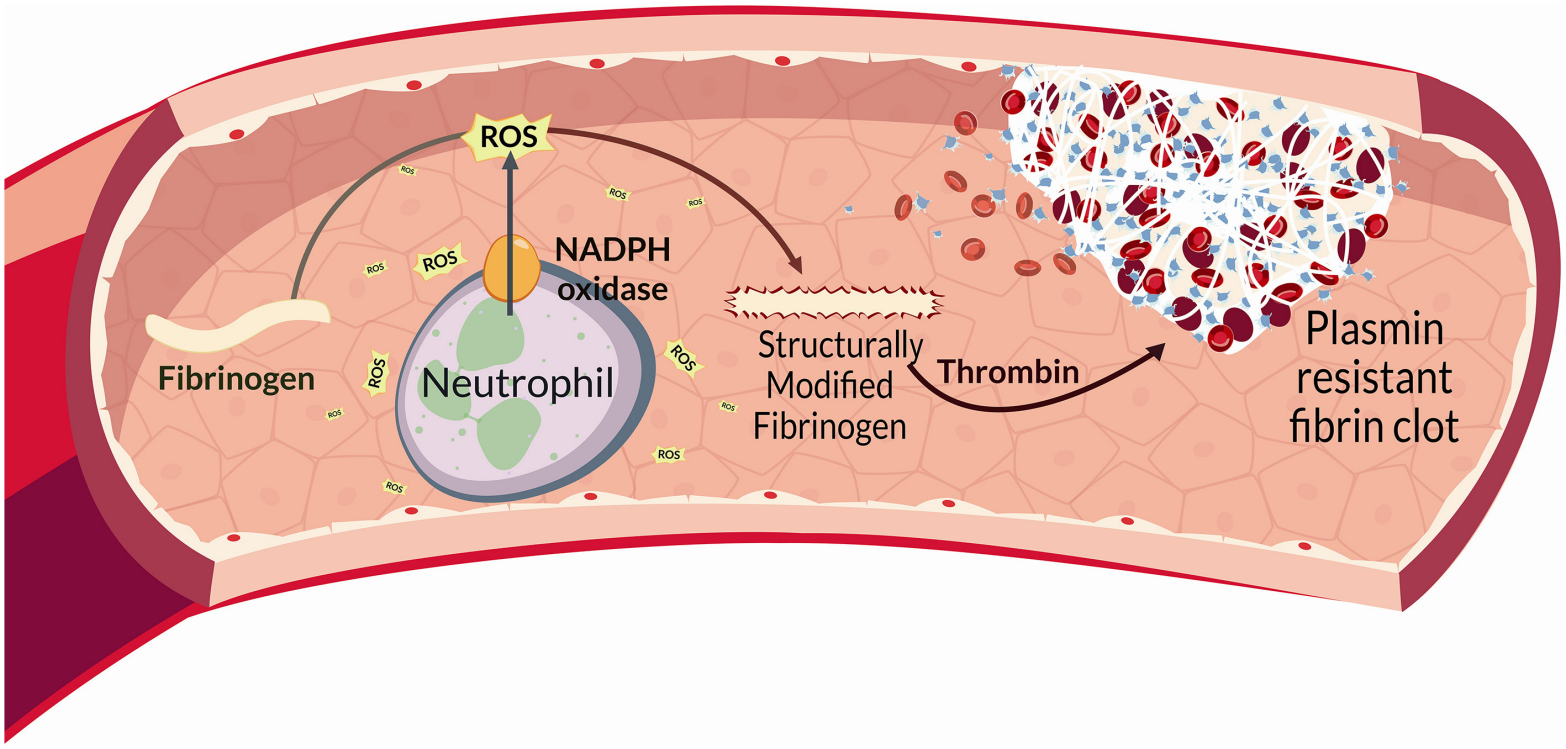
Schematic representation of the NADPH oxidase (NOX2) enzyme complex. NOX2 activation depends on the assembly of four cytosolic proteins (p47phox, p67phox, p40phox, and Rac2) with two transmembrane proteins representing the catalytic core of NOX2 (i.e., p22phox and gp91phox), which form the flavocytochrome b558 complex. In particular, the gp91phox subunit consists of six transmembrane domains, and in its C-terminal region is located the binding sites for FAD and NADPH. The assembly of cytosolic subunits with membrane-bound cyt b558 complex induces the transfer of electrons from cellular NADPH to molecular oxygen and the consequent formation of ${\text{O}}_{2}^{-.}$. Once activated, about 10 nmol/min ${\text{O}}_{2}^{-.}$ per million neutrophils are produced during the oxidative burst.

Finally, clot structure, revealed in BS, a modified architecture mostly characterized by a tight fibrin network composed of filaments with slightly decreased average fiber size that showed a marked resistance to plasmin-induced lysis. This is in line with the findings reporting that clots composed of thin fibers and reduced pores appear more thrombogenic (88, 89). All these features strictly correlate with inflammation and oxidative stress (56, 90, 91) (Figure 2).

Altogether, the above findings indicate that neutrophil activation promotes fibrinogen oxidation and thrombus formation in BS. More specifically, neutrophil ROS via NADPH oxidase are able to modify fibrinogen structure promoting changes in fibrinogen function. Interestingly, all the above data were obtained in a population of patients with inactive disease and regardless of the presence of vascular involvement, thus suggesting that BS represents “*per se*” a model of inflammation-induced thrombosis.

## Author Contributions

GE, MB, and CF conceived the structure of manuscript and drafted the paper. AB, GH, and DP critically revised the manuscript. All the Authors approved the final version of the manuscript.

### Conflict of Interest Statement

The authors declare that the research was conducted in the absence of any commercial or financial relationships that could be construed as a potential conflict of interest.
